# Comparative network stratification analysis for identifying functional interpretable network biomarkers

**DOI:** 10.1186/s12859-017-1462-x

**Published:** 2017-03-14

**Authors:** Chuanchao Zhang, Juan Liu, Qianqian Shi, Tao Zeng, Luonan Chen

**Affiliations:** 1 State Key Laboratory of Software Engineering, School of Computer, Wuhan University, Wuhan, 430072 China; 2 Key Laboratory of Systems Biology, Innovation Center for Cell Signaling Network, Institute of Biochemistry and Cell Biology, Shanghai Institutes for Biological Sciences, Chinese Academy of Sciences, Shanghai, 200031 China

**Keywords:** Network biomarker, Complex disease, Network stratification, Integer programming

## Abstract

**Background:**

A major challenge of bioinformatics in the era of precision medicine is to identify the molecular biomarkers for complex diseases. It is a general expectation that these biomarkers or signatures have not only strong discrimination ability, but also readable interpretations in a biological sense. Generally, the conventional expression-based or network-based methods mainly capture differential genes or differential networks as biomarkers, however, such biomarkers only focus on phenotypic discrimination and usually have less biological or functional interpretation. Meanwhile, the conventional function-based methods could consider the biomarkers corresponding to certain biological functions or pathways, but ignore the differential information of genes, i.e., disregard the active degree of particular genes involved in particular functions, thereby resulting in less discriminative ability on phenotypes. Hence, it is strongly demanded to develop elaborate computational methods to directly identify functional network biomarkers with both discriminative power on disease states and readable interpretation on biological functions.

**Results:**

In this paper, we present a new computational framework based on an integer programming model, named as Comparative Network Stratification (CNS), to extract functional or interpretable network biomarkers, which are of strongly discriminative power on disease states and also readable interpretation on biological functions. In addition, CNS can not only recognize the pathogen biological functions disregarded by traditional Expression-based/Network-based methods, but also uncover the active network-structures underlying such dysregulated functions underestimated by traditional Function-based methods. To validate the effectiveness, we have compared CNS with five state-of-the-art methods, i.e. GSVA, Pathifier, stSVM, frSVM and AEP on four datasets of different complex diseases. The results show that CNS can enhance the discriminative power of network biomarkers, and further provide biologically interpretable information or disease pathogenic mechanism of these biomarkers. A case study on type 1 diabetes (T1D) demonstrates that CNS can identify many dysfunctional genes and networks previously disregarded by conventional approaches.

**Conclusion:**

Therefore, CNS is actually a powerful bioinformatics tool, which can identify functional or interpretable network biomarkers with both discriminative power on disease states and readable interpretation on biological functions. CNS was implemented as a Matlab package, which is available at http://www.sysbio.ac.cn/cb/chenlab/images/CNSpackage_0.1.rar.

**Electronic supplementary material:**

The online version of this article (doi:10.1186/s12859-017-1462-x) contains supplementary material, which is available to authorized users.

## Background

**Fig. 1:**
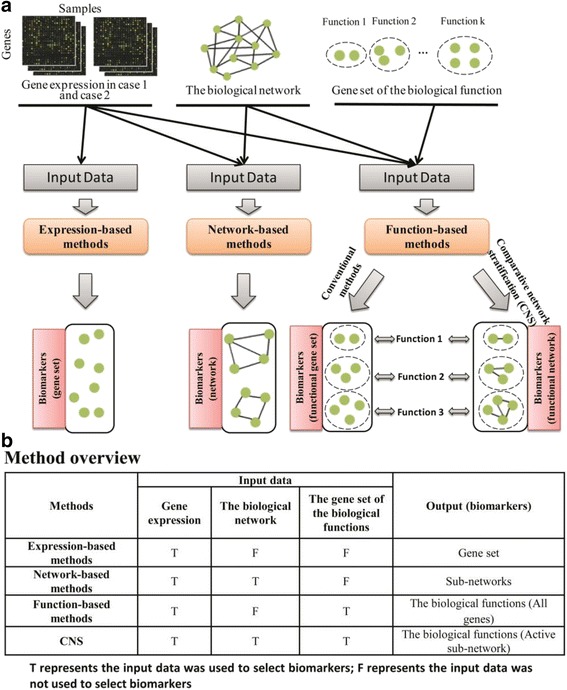
An overview of computational methods for identifying biomarkers. **a** The framework of three kinds of conventional methods; **b** The brief comparison of our and other methods on input and output data

It is of great importance to capture the reliable molecular biomarkers, which is able to accurately diagnose or even predict the relevant clinical characteristics for a new patient with complex diseases [1]. The traditional approaches can be broadly divided into three categories: expression-based, network-based and function-based methods. Expression-based methods, such as SAM [2], obtained some gene sets as the molecular biomarkers according to differential expression pattern between normal and disease samples (Fig. 1). However, cellular heterogeneity within tissues and genetic heterogeneity across patients could weaken the discriminative power of individual genes [3, 4] and then affect the final performance of expression-based methods on independent datasets. Meanwhile, network-based methods, such as frSVM [5] and stSVM algorithm [6], were proposed to extract the active sub-networks as network biomarkers, by considering biological network information (Fig. 1). Clearly, such analysis could make us further interpret the mechanisms of complex diseases at a system level [7].Although network biomarkers pay attention on a growing consensus that complex diseases are mostly contributed by multiple genes through their sophisticated interactions rather than by the individual genes [8, 9], network-based analysis could not directly elucidate the biological or functional roles of the excavated genes/interactions on specific conditions or samples due to the accumulated interaction information on all circumstances. In addition, based on such network-centered idea, function-based methods, such as PEA [10], Pathway activity classification [11], GSVA [12] and Pathifier [13], are developed to obtain functional or interpretable signatures by integrating the biological knowledge (e.g., pathways) of genes into molecular network and expression information [14] (Fig. 1). However, the biological annotation deposited in databases is assembled from different resources or projects on various conditions, which makes it hard to precisely determine the actual states of particular biological functions under a specific condition, e.g. when a disease occurs to a person with certain genetic or epigenetic background.

It is necessary to make biomarkers as a standard tool in the clinical application for precision medicine, which requires biomarkers to have not only discriminative power on samples but also clear biological interpretations [15]. In this work, we develop a novel computational framework, namely Comparative Network Stratification (CNS), to identify functional interpretable network biomarkers using gene expression, gene network and biological function together. Particularly, our biomarkers of the active genes and network structures underlying certain biological functions can better characterize diseases in terms of both discriminative power on phenotypes and readable interpretation on biological functions. To validate the effectiveness of our approach, we have compared CNS with five state-of-the-art methods (such as GSVA, Pathifier, stSVM, frSVM and AEP) on four datasets. The results suggested that CNS can simultaneously identify more discriminative network biomarkers as well as exhibit their biological interpretation in the form of network structure and function annotation. Moreover, we have also applied CNS on a case study of T1D, and provided more biological information on dysfunctional description than other methods. Therefore, CNS is actually a powerful bioinformatics tool, which can investigate functional interpretable network biomarkers in a whole transcriptome and function-centered manner.

## Methods

**Fig. 2:**
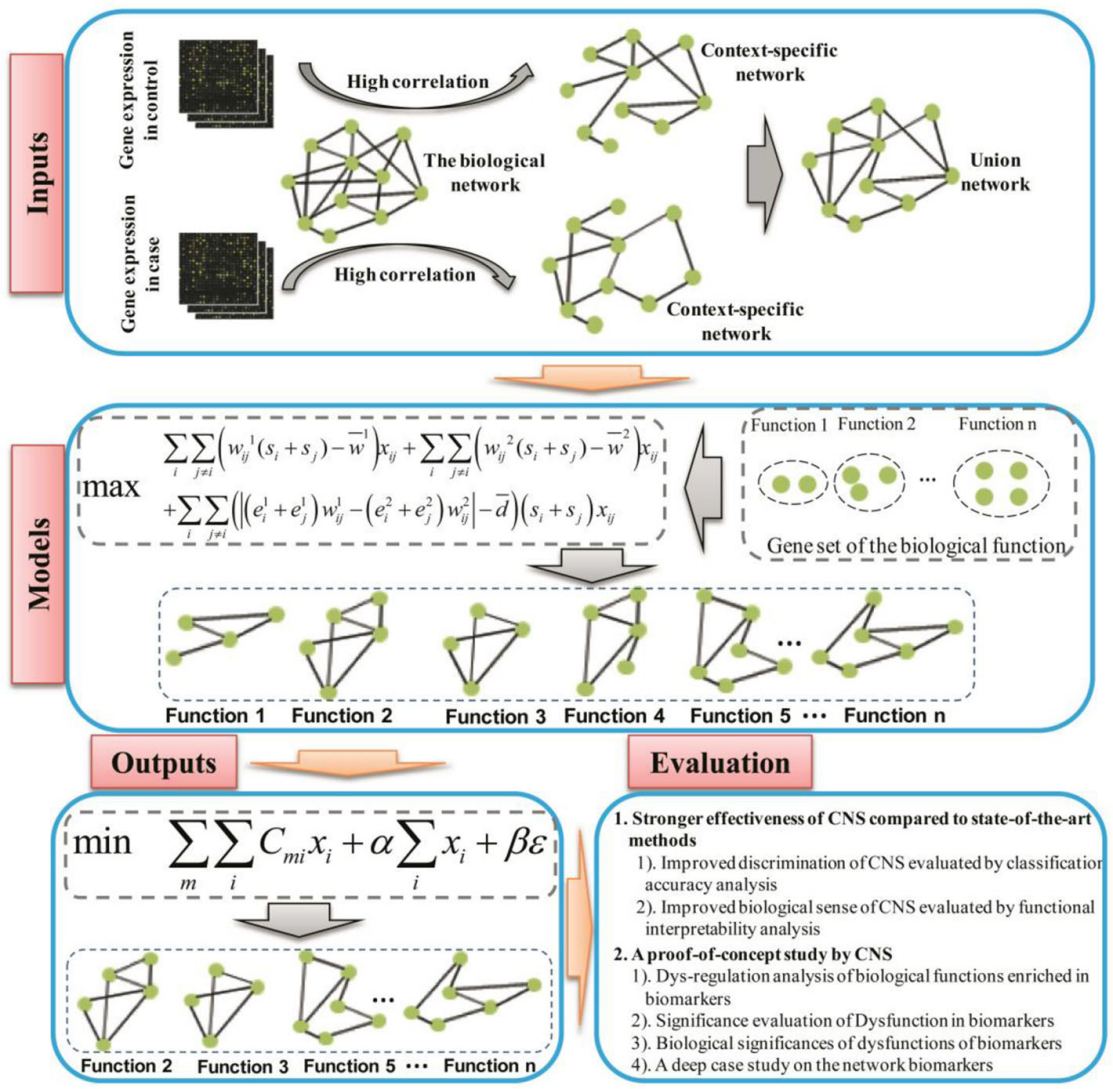
The overview of comparative network stratification (CNS) for identifying functional interpretable network biomarkers

In this section, we describe the computational framework of CNS (Fig. 2). We first introduce data pre-procession, and then present the mathematical model of comparative network stratification to extract networks based on prior-known biological functions. Finally, we apply a classification-based model to select network biomarkers.

### Data pre-procession

Given multiple states (e.g. normal and disease, or disease subtypes), the certain context-specific gene co-expression networks were first constructed before applying our CNS. Patients' expression profiles were mapped onto a biological network obtained from the STRING database (http://string-db.org/), by removing missing genes and keeping those interactions with high Pearson correlation coefficients (*FDR* < 0.01) between gene pairs. These state-specific networks are then integrated as a union background network G used in the following steps.

### Extraction of functional interpretable network

As known, the annotations of genes or proteins from Gene Ontology (GO) are described by structured vocabulary, i.e. GO terms [16, 17]. We use the biological processes (BP) terms as prior-known functional genes' collaboration in this step. Given the above obtained background network G, a mixed integer-programming model (i.e. CNS) determines the sub-network corresponding to a certain GO term (e.g. term *t*), where genes show differential activities in different states (e.g. normal and disease). In other words, an optimal sub-network *F = {N, E}* should be a functional interpretable gene community derived from *G*, subject to

i) *F* should be a sub-network of *G*;
ii) *F* should be a connected graph;
iii) *F* should have enrichment on the genes annotated with GO term *t*;
iv) *F* should indicate the most active alterations between the weighted context-specific network corresponding to different states.

Such an optimization problem can be solved by flux balance process as the formula below:

1 $$ \begin{array}{cc}\hfill {\displaystyle \underset{x,y;o}{ \max }}\hfill & \hfill \begin{array}{l}{\displaystyle \sum_i{\displaystyle \sum_{j\ne i}\left({w_{ij}}^1\left({s}_i+{s}_j\right)-{\overline{w}}^1\right)}}{x}_{ij}+{\displaystyle \sum_i{\displaystyle \sum_{j\ne i}\left({w_{ij}}^2\left({s}_i+{s}_j\right)-{\overline{w}}^2\right)}}{x}_{ij}\\ {}+{\displaystyle \sum_i{\displaystyle \sum_{j\ne i}\left(\left|\left({e}_i^1+{e}_j^1\right){w}_{ij}^1-\left({e}_i^2+{e}_j^2\right){w}_{ij}^2\right|-\overline{d}\right)\left({s}_i+{s}_j\right){x}_{ij}}}\end{array}\hfill \\ {}\hfill s.t.\hfill & \hfill \left\{\begin{array}{l}{\displaystyle \sum_i{x}_{io}=0},o\in \left\{ seed\right\}\\ {}{\displaystyle \sum_j{y}_{oj}}={\displaystyle \sum_i{\displaystyle \sum_{j\ne i}{x}_{ij}}}-{\displaystyle \sum_j{x}_{oj}}\\ {}{\displaystyle \sum_j{y}_{ji}}-{\displaystyle \sum_k{y}_{ik}}={\displaystyle \sum_k{x}_{ik}},\forall i\\ {}{y}_{ij}\le V{x}_{ij},\forall i,j\\ {}{x}_{ij}+{x}_{ji}\le 1,\forall i,j\\ {}{x}_{ij}\in \left\{0,1\right\}\end{array}\right.\hfill \end{array} $$

Here, ${\displaystyle \sum_i}{\displaystyle {\sum}_{j\ne i}}\left({w}_{ij}^1\left({s}_i+{s}_j\right)-{\overline{w}}^1\right){x}_{ij}$ and ${\displaystyle \sum_i}{\displaystyle {\sum}_{j\ne i}}\left({w}_{ij}^2\left({s}_i+{s}_j\right)-{\overline{w}}^2\right){x}_{ij}$ can measure how annotative the selected sub-network is in GO term *t* under two conditions/states respectively. ${\displaystyle \sum_i}{\displaystyle {\sum}_{j\ne i}}\left(\left|\left({e}_i^1+{e}_j^1\right){w}_{ij}^1-\left({e}_i^2+{e}_j^2\right){w}_{ij}^2\right|-\overline{d}\right)\left({s}_i+{s}_j\right){x}_{ij}$describes how great the edge weights of a functional sub-network change. Besides, all the constraint conditions can make sure flux balance so as to meet the selection criteria. In details, *e*_*i*_^1^ and *e*_*i*_^2^ are the average expression values of gene *i* in two states, while *w*_*ij*_^1^ and *w*_*ij*_^2^ are the directional relationship strength of a gene pair or edge (*i* → *j*) in the context-specific networks. For simplicity, Pearson correlation coefficient of gene-pairs (*i* → *j* and *j* → *i*) is used as the edge weight. And ${\overline{w}}^1$ and ${\overline{w}}^2$ represent average edge strength of sub-networks. Similarly, $\overline{d}$ is the average value of all the edge-alterations in network *G*. At the same time, several indicators are also necessarily defined: *s*_*i*_ and *s*_*j*_ are binary (i.e., 0 or 1), representing whether corresponding genes (i.e., gene *i* and *j*) are annotated by term *t* or not, and *x*_*ij*_ is another indicator that *x*_*ij*_ = 1 if the interaction or edge (*i → j*) is selected in a given term, otherwise *x*_*ij*_ = 0.

Noted that the network flux is assumed to originate from a "seed" gene *o* and flow downstream into a bounded sub-network, where any node can be reachable from the seed. In such a connected sub-network, the flux balance could be defined as ${\displaystyle {\sum}_j}{y}_{ij}-{\displaystyle {\sum}_k}{y}_{ik}={\displaystyle {\sum}_k}{x}_{ik}$, where *y*_*ji*_ (different from *w*_*ji*_) represents the value of virtual flow from *j* to *i* and ${\displaystyle \sum_k}{x}_{ik}$ is the out-degree of node *i. V* is a maximum value, which can guarantee that if *x*_*ij*_ is zero, its flow *y*_*ij*_ also equals zero.

### Identification of the functional interpretable network biomarkers

After the above optimization process, we obtained the set of active functional sub-networks corresponding to all GO terms. Thus, a network-based classification model is further proposed to identify the biomarkers from the primary disease-relevant sub-networks, according to the following defined network score.

#### Network score

A quantitative score is required to measure the discriminative ability of an active functional network. Specifically, the network score (NS) of a given sub-network *F* in one sample can be calculated via Eq.(2).

2 $$ N{S}_m=\frac{{\displaystyle \sum_{\left(i,j\right)\in E}\frac{{e_i}_m+{e_j}_m}{2}}}{\sqrt{\left|E\right|}} $$

where *e*_*im*_ and *e*_*jm*_ are the expression values of the nodes/genes *i* and *j* in a sample m when the edge/interaction (*i*, *j*) belongs to *F*; |*E*| is the total number of edges in *F*.

Noted, our *NS* is actually quantified by the expression profiles as well as related to the topology of sub-networks, consistent with the “network activity” definition in previous studies [18–22].

#### Classification-based model

Next, using the NS to assess network activities, a classification-based model can pick out an optimal network biomarker combination [23–25]. One in-house classifier was previously designed to select the minimal number of network features with great classification capacity [20]. Here, we extended this mathematical model to achieve ‘elastic’ classification by adding a correct regulization. Such a modified model is formulated as below:

3 $$ \begin{array}{l} \min \kern1.5em {\displaystyle \sum_m{\displaystyle \sum_i{C}_{mi}{x}_i}}+\alpha {\displaystyle \sum_i{x}_i}+\beta \varepsilon \\ {}\kern3.6em C{\left({x}_1,{x}_2,\cdots, {x}_n\right)}^T\le \varepsilon \\ {}s.t\kern2.5em {\displaystyle \sum_i{x}_i}\ge 1\\ {}\kern3.5em \varepsilon >=0\\ {}\kern3.3em {x}_i\in \left\{0,1\right\}\end{array} $$

where *x*_*i*_ is binary (i.e., 0 or 1), indicating whether the sub-network *i* is selected or not; And C is a function matrix, where each element *C*_*ij*_ representing *j*_th_ network's contribution to *i*_th_ sample if the sample is assigned into the correct group [20]. *ε* is the 'elastic' correct regulator with its value as small as possible.

In the objective function, the first term is used to characterize the classification capacity of selected biomarkers; the second term is to minimize the marker number in selection process; and the third term is to minimize the classification error; *α* and *β* are positive penalty parameters to control the trade-off within signature number, classification err and classification capacity. Certainly, *α* and *β* are chosen to obtain the best classification ratio by tuning in a reasonable scale.

In the constraint array, the first constraint is used to ensure an acceptable sample classification; the second constraint is used to guarantee at least one functional sub-network should be selected as final biomarker; and the third constraint is used to generate a reasonable correction in practice.

## Results

### Stronger effectiveness of CNS compared to state-of-the-art methods

To validate the effectiveness of CNS, a complete comparison scheme had been built to evaluate the performances of the conventional biomarker discovery methods and CNS on gene expression datasets. There are four datasets used in the comparison, i.e. GSE38642 (54 normal vs. 9 disease) [26], GSE18732 (47 normal vs. 45 disease) [27], GSE27342 (80 normal vs. 80 disease) [28] and GSE35713 (79 normal vs. 57 disease) [29]. The compared methods include GSVA [12], Pathifier [13], stSVM [6], frSVM [5] and AEP [10]. As stSVM, frSVM and AEP have been integrated into the netClass package [1], we used the netClass package directly. Due to GSVA and Pathifier implemented without feature selection, we select the same number biomarkers as identified by CNS through SVM-RFE [30].

As proposed, good biomarkers or signatures should have more discrimination ability as well as more interpretable biological sense. Thus, we used two criterions to evaluate the identified biomarkers (see Additional files 1, 2, 3 and 4) respectively: classification accuracy and functional interpretability.

#### Improved discrimination of CNS evaluated by classification accuracy analysis

**Fig. 3:**
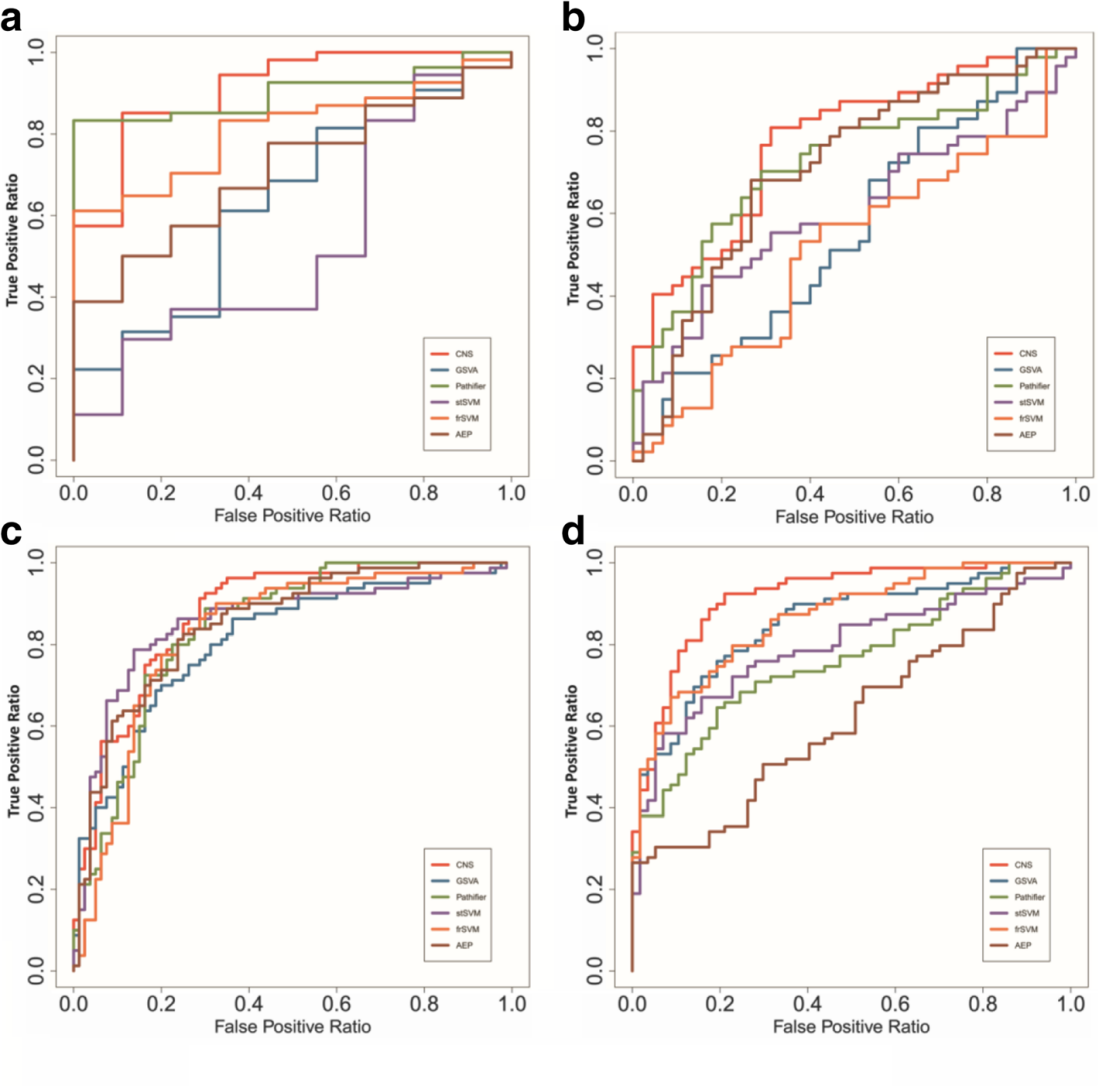
The performance of all compared methods across multiple datasets in classification accuracy analysis. The ROC curves of the compared methods on **a** GSE38642, **b** GSE18732, **c** GSE27342, **d** GSE35713 datasets

We used SVM to calculate the classification accuracy of the biomarkers identified by all methods, employing five-fold cross-validation. The performance of different methods on four datasets were shown as ROC curves (Fig. 3). And the AUC corresponding to these ROC curves were reported in Table 1. These results illustrate CNS biomarkers have the most stable and best classification accuracy than those identified by other methods. Note that, we can see, in the low false positive regions, the true positive rate of CNS is lower than some of other methods, that might be caused by the trade-off between specificity and sensitivity of classification approaches. It would be valuable to further improve the accuracy by careful feature selection or classifier building in future work.

**Table 1:** The AUC of six methods on four datasets

Methods	GSE38642	GSE18732	GSE27342	GSE35713	Mean ± SD
CNS	0.911	0.777	0.885	0.916	0.872 ± 0.06
GSVA	0.637	0.561	0.802	0.851	0.712 ± 0.13
Pathifier	0.9012	0.726	0.848	0.761	0.809 ± 0.08
stSVM	0.528	0.603	0.855	0.792	0.694 ± 0.15
frSVM	0.812	0.513	0.827	0.865	0.754 ± 0.16
AEP	0.711	0.708	0.852	0.621	0.723 ± 0.09

#### Improved biological sense of CNS evaluated by functional interpretability analysis

In order to further evaluate the interpretability of these methods on biological functions, we made an association analysis to measure the relationship between the biomarkers and the studied disease, as the functional interpretability power of those biomarkers. And the association degree is evaluated by the proportions of disease-associated genes (DAGs) in all selected functional biomarkers. Here, the DAGs are the intersection of the differential expressed genes (DEGs) identified by *t*-test and genes associated with the studied disease taken from GeneCard [31]. Meanwhile, the biomarkers identified by frSVM and stSVM are general gene sets rather than functional gene groups by GSVA, Pathifier and AEP; that’s why we first functionally label them with the top enriched terms by g:Profiler [32].

**Fig. 4:**
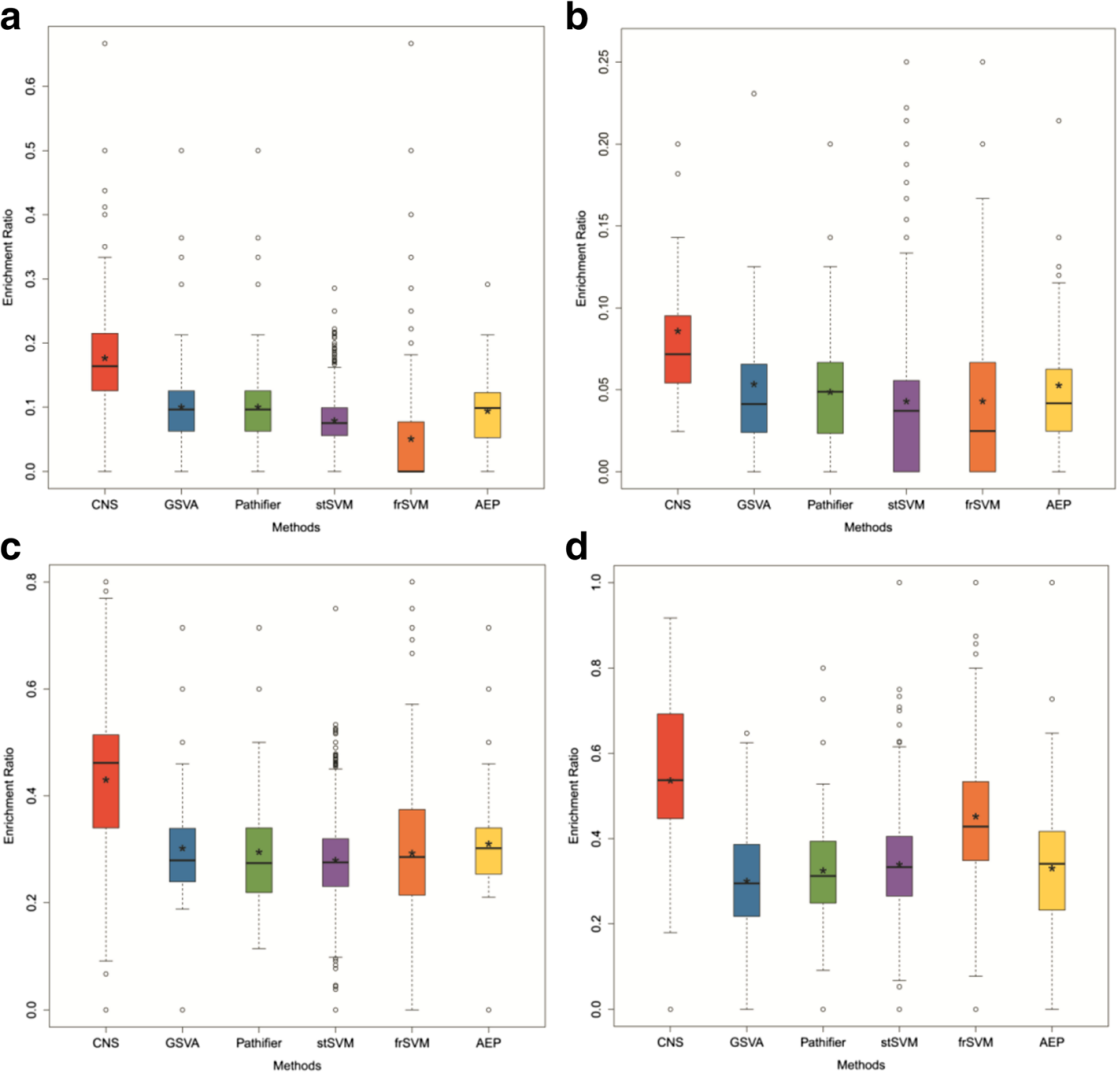
The performance of all compared methods across multiple datasets for functional interpretability analysis. The “*” represents the mean value of the functional interpretability power for each method and “-” indicates the median value. The functional interpretability analysis of the compared methods on **a** GSE38642, **b** GSE18732, **c** GSE27342, **d** GSE35713 datasets respectively

The enrichment ratio boxplots are shown in Fig. 4. It seems CNS has the best performance on every dataset; frSVM has varied performance than other conventional approaches showing its dependency on the context of analyzed datasets; while other methods have nearly the same performances across all datasets. This fact suggests many previous methods would consider less on the functional interpretability, so that they tend to have the similar lower performances; and CNS actually promote the interpretability of identified biomarkers on biological functions as proposed.

Furthermore, we also computed the *P*-values to measure CNS improved performance compared to other methods, and shown in Table 2. Obviously, CNS has much better performance on the functional interpretability overall.

**Table 2:** The significance of better performance on functional interpretability comparison

Datasets	CNS vs. GSVA	CNS vs. Pathifier	CNS vs. stSVM	CNS vs. frSVM	CNS vs. AEP	<*P*-value
GSE38642	4.67E-17	1.43E-15	7.65E-92	1.34E-74	5.41E-04	1.0E-04
GSE18732	0.0183	1.29E-04	6.92E-08	6.40E-05	0.015	0.02
GSE27342	1.11E-05	1.52E-05	3.60E-20	5.49E-08	1.33E-05	1.0E-05
GSE35713	5.61E-15	5.46E-13	2.05E-44	1.14E-06	9.12E-16	1.0E-15

### A proof-of-concept study by CNS

The biomarkers identified by CNS not only determine specific biological functions related to diseases, but also reveal the active network-structure underlying such dysfunctions. These characteristics of biomarkers just make that CNS have improved ability to understand complex diseases. To further illustrate this advantage of CNS, we have made a proof-of-concept study on GSE3571 dataset of T1D (i.e. 79 normal samples and 57 T1D samples).

#### Dys-regulation analysis of biological functions enriched in biomarkers

**Fig. 5:**
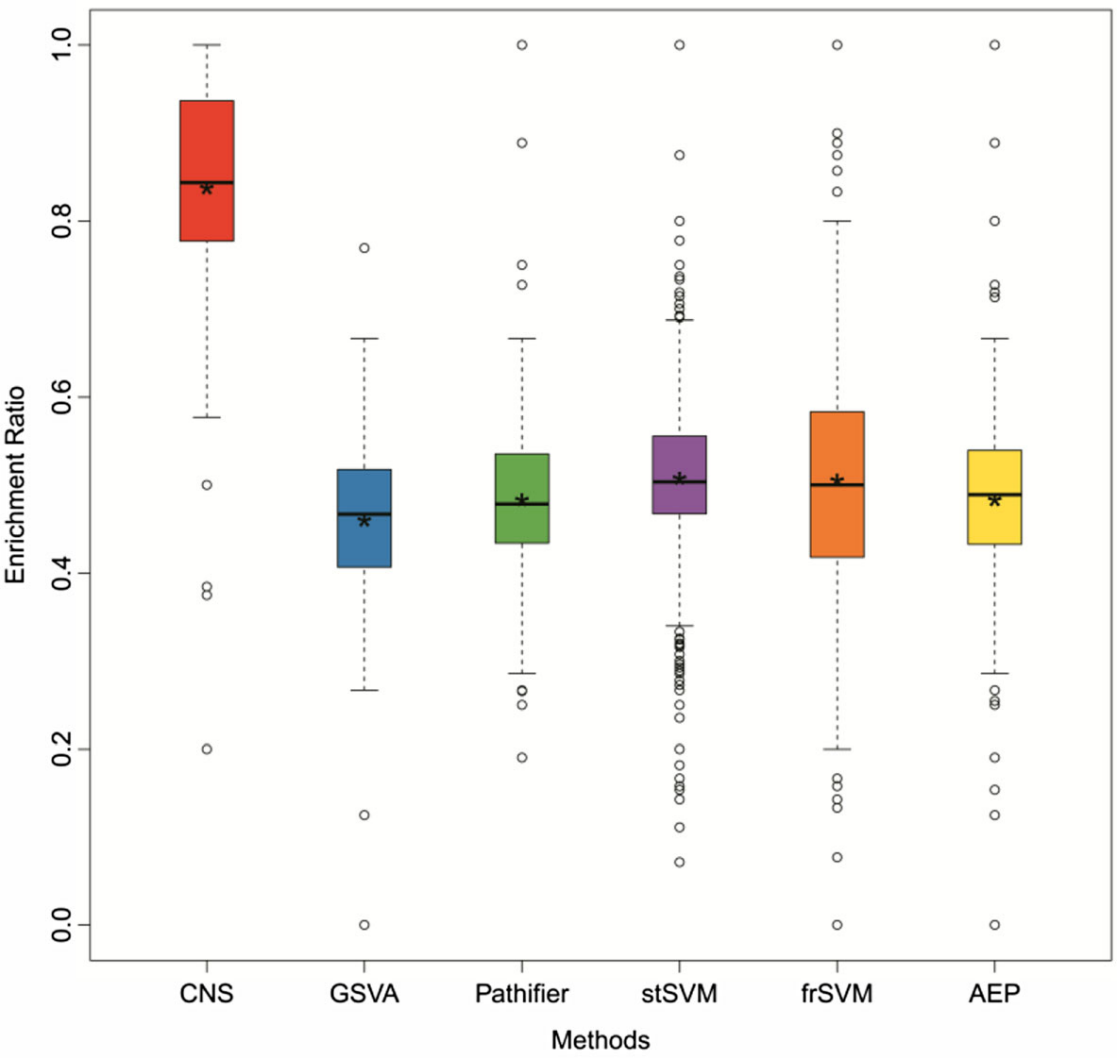
The performance of the compared methods for dysfunction analysis. The “*” represents the mean value of the functional interpretability power for each method and “-” indicates the median value

Biomarkers would play important roles on the development and progression of complex diseases, and they would be dysfunctional among different disease states. The DEGs proportion is regarded as an effective standard to estimate the dysfunction degree of the biomarkers. Thus, the enrichment ratios of DEGs in each biological functions were calculated to represent the dysfunction degree of our biomarkers. The results were shown in Fig. 5, and CNS obtains the biomarkers with significant dysfunctional signal again.

On one hand, the traditional function-based methods (GSVA, Pathifier and AEP) and CNS all selected function biomarkers. The traditional methods assumed to consider all genes of each biological functions and transform gene expressions to some meta-values with good distinguishable capacity. Different to them, CNS recognized the representative genes of each biological function, which would promote the enrichment of DEGs, and reflect more reasonable dysfunction interpretation.

On the other hand, the traditional expression-based and network-based methods are designed to identify gene sets or sub-networks as biomarkers. Although these biomarkers could contain many DEGs, these enriched genes could be isolated and cannot provide readable functional interpretation. According to these results of DEGs enrichment for biomarkers, the performance of the traditional expression-based and network-based methods may be slightly better than traditional function-based methods, but still be less than CNS.

#### Significance evaluation of dysfunction in biomarkers

Besides dysfunction degree measurement, it is also necessary to evaluate the level of significance of those dysfunctions in the biomarkers. A hyper-geometric test is used to compute the *P*-value of the enrichments of DEGs for a biomarker/a biological function.

4 $$ P=1-{\displaystyle \sum_{k=0}^{X-1}\frac{\left(\begin{array}{c}\hfill G\hfill \\ {}\hfill k\hfill \end{array}\right)\left(\begin{array}{c}\hfill R-G\hfill \\ {}\hfill T-k\hfill \end{array}\right)}{\left(\begin{array}{c}\hfill R\hfill \\ {}\hfill T\hfill \end{array}\right)}} $$

where R is the number of all genes, T is the number of genes in the biomarker/the biological function; G is the number of DEGs; X is the number of DEGs enriched in the biomarker/the biological function.

**Fig. 6:**
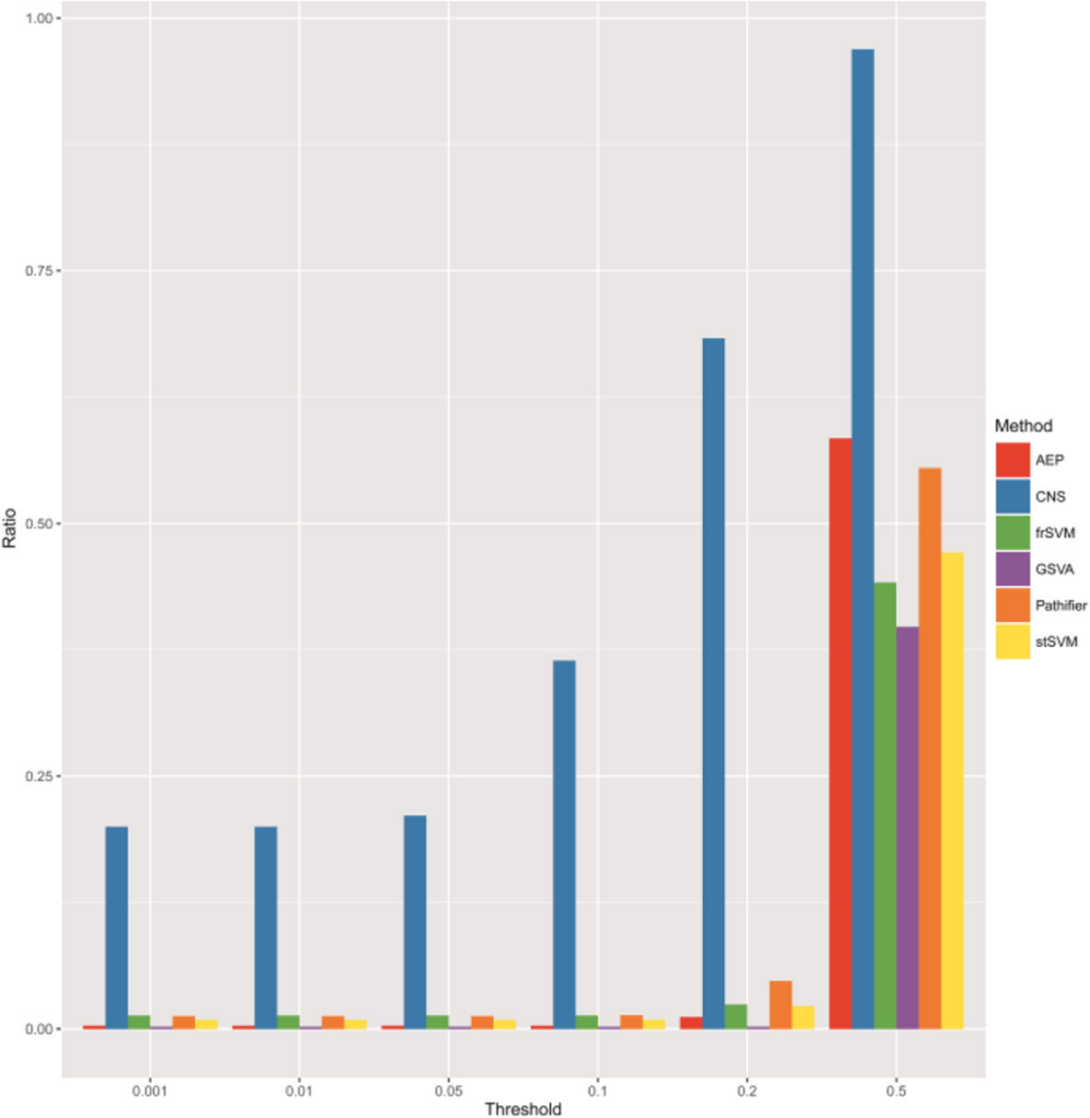
Percentages of dysfunctions obtained under different thresholds of significance

Given a threshold of statistic significance, we define those biomarkers/biological functions with less *P*-values as significant ones. And then the ratios of these significant biomarkers/functions in identified biomarkers under the varied thresholds are shown in Fig. 6. Obviously, CNS is more effective to obtain dysfunction-explainable biomarkers than other methods.

#### Biological significances of dysfunctions of biomarkers

The most significant dysfunction of ten biomarkers/functions identified by CNS are listed in Table 3, five of which are related to T1D as reported in literatures. These biomarkers include: two functions direct correlated with T1D (e.g. regulation of insulin secretion and regulation of gluconeogenesis [31]), and three functions relevant with T1D complications (e.g. positive regulation of cytokine-mediated signaling pathway [33], positive regulation of response to cytokine stimulus [34, 35], establishment of T cell polarity [35–37]).

**Table 3:** The biological sense of dysfunction of biomarkers

Biomarkers Name	*P*-values_Dis	*P*-values_Diff	R
positive regulation of cytokine-mediated signaling pathway	1.42E-09	1.36E-05	Y
positive regulation of response to cytokine stimulus	3.5E-03	9.79E-06	Y
lipid metabolic process	5.11E-04	4.34E-05	N
positive regulation of osteoclast differentiation	6.79E-04	1.95E-06	N
regulation of insulin secretion	9.95E-11	6.28E-10	Y
beta-amyloid clearance	5.71E-09	8.51E-05	N
regulation of gluconeogenesis	1.15E-08	6.5E-07	Y
establishment of T cell polarity	1.7E-08	1.9E-10	Y
epithelial cell differentiation	3.68E-08	2.4E-05	N
cellular lipid metabolic process	3.52E-08	3.25E-05	N

*P*-values_Dis evaluates the significance of the disease genes enriched in biomarkers; *P*-values_Diff evaluates the significance of DEGs enriched in the biomarkers; R represents whether one biomarker is known related with T1D or its complications; Y denotes the biomarker is known associated with T1D or its complications; N denotes the biomarker is unclearly associated with T1D

#### A case study on the network biomarkers

Insulin secretion dysfunction is known to be a key characteristic of T1D. And such regulation function identified in our biomarkers is further analyzed to see how its topological structure changes and gene expression perturbations.

**Fig. 7:**
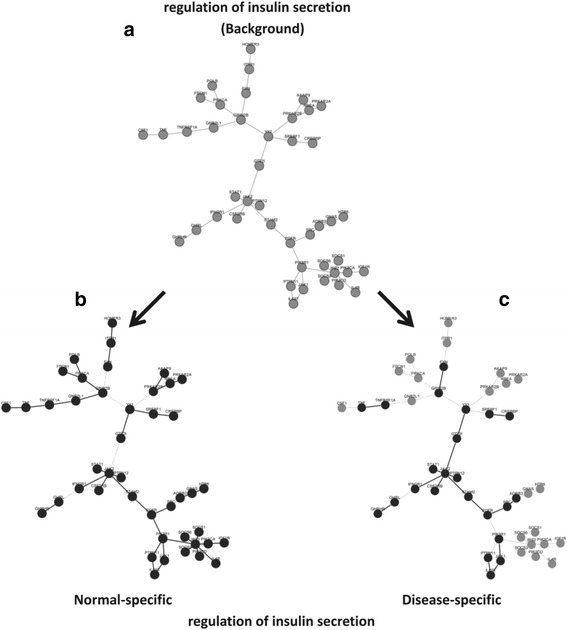
The topology alterations of the network biomarker. **a** The background network is the biomarker of “The regulation of insulin secretion”, which is a union network of (**b**) normal-specific active sub-network and (**c**) disease-specific active sub-network identified by CNS. Nodes and edges in black present genes and interactions are selected under a certain state; Nodes and edges in gray mean genes and interactions are background features and not selected

The overall and state-specific network structures of “the regulation of insulin secretion” were shown in Fig. 7. The network topology under normal condition is very similar to background network structure, while the network under T1D loses much functional completeness (Fisher’s Exact Test *P*-value is 7.31E-06). In particular, as Nils Billestrup et al. ever reported, SOCS inhibited IRS in diabetic patients [38], which agreed with their disappearance in our T1D active network structure. Meanwhile, our network biomarker has 46 genes including 39 DEGs, which means that the network biomarker can represent an active functional module/unit under different states. But when we check the same function in the biomarkers identified by other five methods, the real active genes are more likely drown out in a huge gene set. The function of “the regulation of insulin secretion” is probably missed by network-based methods, because the frSVM biomarkers (94 genes) and stSVM biomarkers (445 genes) have only 4 and 14 genes enriched in this biological function. Besides, for those function-based methods (GSVA, Pathifier and AEP), the real active network structure of this regulation function is also incomplete in their biomarkers, where only 23 DEGs are covered in 55 genes. All the comparison results further show CNS, different from traditional methods, can recognize the representative genes of biological functions, which would promote the enrichment of differential expressed genes, and reflect more readable interpretation on biological dysfunctions.

## Discussion and Conclusion

The complex diseases are usually thought to be caused by the dysfunction on the molecular interaction network (e.g. protein-protein interactions). Conventional expression-based and network-based methods widely use genes and their networks to capture the biomarkers without clear complete biological interpretation due to subsequent function enrichment in the whole gene pool. While, the function-based analysis just solved this problem by integrating biological functions (e.g. pathways or GO terms) into gene sets at the beginning. However, rather than a list of disease associated functions, detailed alterations (e.g. topological structure) in such functions clearly are more valuable to precision medicine study to some extent.

With respect to the problems, we proposed a novel computational framework, named as comparative network stratification (CNS), to identify active sub-networks as functional interpretable network biomarkers, which have both discriminative power on disease states and readable interpretation on biological functions. Actually, we previously also developed a method as Network Stratification Analysis (NetSA) [39] to decompose an context-specific biological network into many function-specific network modules. CNS analyzed the characteristics of two or more states of complex diseases based on the same background biological network. Using CNS, the extended method of NetSA, we tessellated two-states' context-specific networks and identified the active network structures of biological functions under normal and disease conditions, respectively.

To validate the effectiveness of such a new approach, we have compared CNS with five state-of-the-art methods (such as GSVA, Pathifier, stSVM, frSVM and AEP) on four datasets of complex diseases. These results demonstrate CNS considers the representative genes and their networks in biological functions, and thus it can have better discrimination, better enrichment on disease-associated genes and better enrichment on differential expressed genes simultaneously. Therefore, CNS will be actually a powerful bioinformatics tool to investigate functional interpretable network biomarkers in a whole transcriptome and function-centered manner.

Besides, we mainly focus on the discrimination between normal and disease, which can be expanded to distinguish multiple diseases in future work. In fact, in our experiments, we have analysis on type 1 diabetes (GSE35713) and type 2 diabetes (GSE38642) in this paper. As known to us, type 1 diabetes (T1D) and type 2 diabetes (T2D) are two subtypes of Diabetes and have similar disease genes from the GeneCard database including 5066 and 4970 disease genes, respectively. However, the number of the identified biomarkers of T1D and T2D are 91 and 164 respectively, and the overlap of them only contains 7 biomarkers. Meanwhile, Figs. 4d and a both show the functional interpretability of the identified biomarkers of T1D and T2D respectively. Based on these results, we could find that the functional interpretability of the identified biomarkers in T1D and T2D is indeed different, and this fact suggests that the different genes or functions form the different biomarkers for T1D and T2D. Therefore, the identified functional biomarkers are sensitive and specific on the diseases.

In addition, considering the functional containment relationships or the ancestors-descendants relationships of the biological functions in GO database, it is necessary to remove redundant functional interpretations in the network biomarkers in the future work around CNS.

## Additional files

Additional file 1: biomarker-GSE18732.xlsx, the identified biomarkers of GSE18732 dataset. (XLSX 40 kb)

Additional file 2: biomarker-GSE27342.xlsx, the identified biomarkers of GSE27342 dataset. (XLSX 30 kb)

Additional file 3: biomarker-GSE35713.xlsx, the identified biomarkers in GSE35713 dataset. (XLSX 116 kb)

Additional file 4: biomarker-GSE38642.xlsx, the identified biomarkers in GSE38642 dataset. (XLSX 207 kb)
